# Development of a quality of life questionnaire for patients with pancreatic neuroendocrine tumours (the PANNET module)

**DOI:** 10.1111/jne.13097

**Published:** 2022-02-13

**Authors:** John K. Ramage, Elizabeth Friend, Jordan Randell, Barbara KING, Paz Fernandez Ortega, Mairéad G. McNamara, Gregory Kaltsas, Massimo Falconi, Jaroslav Cwikla, Jaume Capdevila, Simona Grozinsky‐Glasberg, Dalvinder Mandair, Eva Gamper, Raj Srirajaskanthan, Martin O Weickert, Debra Gray, the EORTC Quality of Life Group

**Affiliations:** ^1^ 9946 Hampshire Hospitals NHS Foundation Trust Basingstoke UK; ^2^ 8629 University of Winchester Winchester UK; ^3^ Instit Catala d’Oncologia Barcelona Spain; ^4^ Division of Cancer Sciences University of Manchester/The Christie NHS Foundation Trust Manchester UK; ^5^ National and Kapodistrian University of Athens Athens Greece; ^6^ Pancreatic Surgery Unit Pancreas Translational & Clinical Research Center IRCCS San Raffaele Scientific Institute Milan Italy; ^7^ Indywidualna Specjalistyczna Praktyka Lekarska Warsaw Poland; ^8^ Vall D’Hebron University Hospital Vall Hebron Institute of Oncology (VHIO) Barcelona Spain; ^9^ Hadassah‐Hebrew University Medical Centre Jerusalem Israel; ^10^ 4965 Royal Free London NHS Foundation Trust London UK; ^11^ 27280 Medical University of Innsbruck Innsbruck Austria; ^12^ Kings Health Partners Neuroendocrine Centre London UK; ^13^ Arden NET Unit Coventry UK

**Keywords:** gastrinoma, insulinoma, pancreatic neoplasm, pancreatic neuroendocrine tumour, quality of life

## Abstract

Pancreatic neuroendocrine tumours (panNET) are heterogeneous neoplasms usually characterised by slow growth and secretion of hormones, which often cause symptoms. The effect of these symptoms on quality of life (QoL) has not previously been examined in detail. EORTC (European Organisation for Research and Treatment of Cancer) guidelines were followed in phases 1–3 to produce a potential module of questions usable for trials in panNET, focusing on three common types of panNET. For two less common types, a list of symptoms was constructed. Following an extensive literature search and phase 1a interviews with patients and healthcare workers, a long list of potential issues (169) was obtained. This list was shown to 12 patients from three countries in phase 1b interviews to check that no items were missed. The list was reduced to 57 issues. The list of issues was converted to questions, mainly from existing validated questions within the EORTC item library. The list of questions was then used in a phase 3 international study in eight countries using seven languages. A provisional module of 24 items is presented for use in nonfunctioning panNET, gastrinoma and insulinoma. This module increases knowledge concerning QoL in this condition and may be a useful adjunct in clinical trials. A phase 4 trial is being considered for validation of this questionnaire.

## Graphical abstract

The quality of life aspects of pancreatic NET patients have been collected in great detail. The most important aspects have been condensed into a list of 21 questions, to be used with the C30 generic questionnaire. This can be used in trials as well as clinical assessments of these patients.

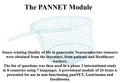

## 1 INTRODUCTION

Neuroendocrine tumours (NETs) are a heterogeneous group of rare neoplasms that arise from many different organs in the body. NETs arising in the gastrointestinal tract are the most common, accounting for two thirds of all NETs.^1^ Specifically, pancreatic NETs (panNET) have an incidence of ≤1 case per 100,000 population per year and account for 12% of all pancreatic tumours.^2^, ^3^, ^4^

Pancreatic NETs can be classified as functioning or nonfunctioning (NF), and the presentation of a panNET is dependent on its functionality.^5^ Functioning panNETs secrete a predominant hormone which determines the hormonal syndrome. In contrast, nonfunctioning (NF) panNETs do not present clinically with a hormonal syndrome, but often with symptoms of local compression or metastatic disease.^6^ Insulinomas and gastrinomas are the most common functioning panNETs, whilst glucagonoma, vasointestinal peptide (VIP)oma, pancreatic polypeptide (PP)oma, somatostatinoma and adrenocorticotropic hormone (ACTH secreting panNETs are rarer. The secreting hormone makes each subtype of panNET present differently, meaning not only are panNETs distinct from other gastrointestinal (GI) NETs, but it is also important to consider the differences in presentation within NETs of pancreatic origin.^7^

The EORTC QLQ‐GINET21 module was fully validated and published in 2013.^8^ This module, used with the QLQ‐C30, is useful for a broad evaluation of QoL in clinical studies of patients with various GINET primaries. However, as panNETs can be associated with distinct hormonal symptoms, and are prognostically different from NETs of gut origin, some clinically pertinent patient reported outcomes may be missing from the QLQ‐GINET21 for patients with panNETs. Furthermore, therapeutic advances in the 14 years since the original development of the GINET module,^9^ may have altered the issues affecting panNET patients. There is a need for a module specifically designed to be used with patients with NETs of pancreatic origin. Given this, the aim of this research was to develop a module that captured QoL issues specifically important to patients with pNETs, as well as considering the differences between panNET subtypes (NF, insulinoma, gastrinoma, VIPoma and glucagonoma).

## 2 MATERIALS AND METHODS

The EORTC Guidelines for Developing Questionnaire Modules (phases 1–3) were followed.^10^ Patient inclusion/exclusion criteria are shown in Appendix A. The research was conducted in the following phases.

### 2.1 Phase 1

A systematic review of the literature addressing symptoms and QoL issues related to pancreatic NETs was conducted in order to generate a preliminary list of issues for five subtypes of panNETs: nonfunctioning, insulinoma, gastrinoma, VIPoma and glucagonoma. The systematic review was published previously.^7^

Following this, phase 1a interviews were conducted with 23 patients with panNET: eight nonfunctioning, seven insulinoma and eight gastrinoma from the UK, Cyprus, Spain, and Italy. This included 14 male participants and nine female participants aged 33–71 years. Healthcare professional interviews were conducted with six clinicians and nine nurses specialising in panNETs from Spain, Italy, Cyprus, and the UK. After the creation of a provisional list, a further set of phase 1b interviews were conducted with 12 patients with panNET: four nonfunctioning, five gastrinoma, and three insulinomas. The patients were recruited from the UK, Italy, and Spain.

During the patient interviews, it became clear that there were not enough patients with VIPoma and glucagonoma to take these subtypes to full module development due to their rarity. We did, however, conduct 18 phase 1 interviews with these rarer patient groups: 10 with VIPoma and eight with glucagonoma. These are reported separately below to provide some insight into quality‐of‐life issues associated with these two rare tumour types. All qualitative data in this phase were analysed using qualitative content analysis^11^ in NVivo 12.

### 2.2 Phase 2

For the remaining subgroups of NF, gastrinoma and insulinoma, the issues were “operationalised” into a set of provisional questions at the end of phase 1. The resulting 57 provisional questions were then taken into phase 3.

### 2.3 Phase 3

For phase 3, 59 patients were recruited from the UK, Northern, Central and Southern Europe as well as one centre outside Europe (Israel). Numbers recruited were: UK 15, Italy six, Spain seven, Poland 10, Austria seven, Israel eight, Greece five, Cyprus one (Appendix B). Patients with a histologically‐confirmed panNET (nonfunctioning, gastrinoma, insulinoma) were considered for this study at any stage in their disease history (e.g., attending an outpatient clinic; inpatient setting receiving treatment; post treatment follow‐up or having a routine surveillance consultation). Basic demographic data and clinical data was extracted from the clinical notes and/or obtained from the patient directly. Patients who participated in phase 1 could not participate in phase 3. All patients were invited to participate in line with the ethical and governance requirements of each participating country.

Patients were asked to complete the QLQ‐C30 general cancer questionnaire as well as the provisional panNET module questionnaire, containing all 57 items from phase 2. There were six translations of the questions, from the English version, performed by the EORTC translation unit.

Patients were asked to answer each question in two ways:

1. According to the extent that they have experienced these symptoms or problems (experience)
2. According to how relevant/important they think each item was to the disease (relevance)

All data was collected using a four‐point Likert response scale (as per guidelines). Quantitative results were calculated using SPSS version 26. Reliability analysis was performed on the final questions. To determine the reliability of the scales, Cronbach's α were computed for relevancy as well as the reliability if each item were deleted.

## 3 RESULTS

### 3.1 Phase 1 and 2 results for patients with nonfunctioning panNET, insulinoma, and gastrinoma

As previously published^7^ a search of the literature generated a total of 4,723 documents. Thus, 71 studies were eligible for data extraction and 141 QoL issues and symptoms of panNETs were extracted from the literature by coding on NVivo software. Duplicates, test results and medical terms (as opposed to symptoms) were removed from the list following review and the list was reduced to 87 issues.

Phase 1a patient interviews identified 156 issues. The list of issues mentioned by patients was combined with the literature search issues and the issues mentioned by healthcare professionals (HCPs). EORTC guidelines were followed in reducing the issue list.^10^ This resulted in a list of 169 issues mentioned by patients and HC professionals; 96 of which were additional to those from the literature search. Two persons in the research group (Gray and Randell) then thematically grouped these issues. At this stage, the team devised exclusion criteria as per guidelines^10^ to reduce the number of issues to 78. Following phase 1b interviews, a further 21 issues were removed, resulting in a provisional item list of 57.

To convert the issues into questions, the EORTC item bank was searched. There were 35 existing questions in the Item Bank and 10 item bank questions were adjusted (words deleted or added). For the remaining 12 issues, two of the authors (Friend and Ramage) reviewed the context of the issues from the qualitative interview data to ensure the emphasis was correct. The resulting questionnaire had 57 provisional questions. The questions were assembled in groups (based on clinical knowledge of items measuring same construct).

### 3.2 Phase 3 results for module development for NF panNET, insulinoma and gastrinoma

Phase 3 data was analysed, and the module team, in accordance with the suggested cutoff criteria outlined in the EORTC Quality of Life Group Guidelines for Developing Questionnaire Modules,^10^ reviewed the mean relevance and experience scores for all 57 items. All items in the provisional panNET module scored more than 1.5 on average, had a percentage ratio of more than 30%, were consistent across cultures and more than 95% of patients responded to every item. Ten items were excluded because responses of 3 “quite a bit” or 4 “very much” occurred for less than 50% of the sample, and two items were excluded because the range of scores was not greater than two, and they also showed ceiling effects (less than 10% of respondents scored either 1 “not at all” or 2 “a little” for these items). Thus, 12 items in total were excluded at the end of this review phase.

The remaining 45 items were then reviewed by the module team on an item‐by‐item basis, in terms of their overall relevance (either to all patients or to a subtype), their clinical significance as a symptom and/or their specificity. This phase resulted in the exclusion of another 21 items. Reasons for exclusion of the 33 items (12 + 21 above) are given in Appendix C. Comparison with the GINET21 module showed overlap in only three questions. These were bloating (Item 9); losing weight (Item 12) and acid indigestion (Item 13).

### 3.3 Resulting module

During the item selection phase, it became apparent that patients with different subtypes of panNET had different experiences and resultant item relevance scores to some questions. Some questions appeared nonspecific and related to all possible patients with panNET (10 questions). Of the remaining questions, we could not easily separate scores in patients with gastrinoma from those who had nonfunctioning tumours. Patients with insulinoma had quite different scores (for example the top three questions scored in insulinoma were low blood sugar, palpitations and sweating, none of which scored highly in the other groups). On this basis, the module team made the decision to create two adapted modules with different questions for different panNET subtypes: one for those with gastrinoma and NF together and one for those with insulinoma.

A flowchart for module development is shown in Appendix E.

The final pNET module therefore includes 10 questions for all patients, five additional questions for nonfunctioning and gastrinoma (15 in total for this subtype) and nine additional questions for those with insulinoma (19 in total for this subtype). The final module is presented in Table 1. Hypothesised subscales were suggested purely on clinical grounds. The Insulinoma questions are clearly mainly related to low blood glucose and there were seven relating to neurological impairment, with two “other” insulinoma questions. These are all mainly specific to insulin secretion and would not overlap with other panNETs.

**TABLE 1:** Retained items with mean scores, the hypothesised subscales and whether the items are for all patients, or those with gastrinoma/nonfunctioning tumours, or insulinoma

Original item number	Item	Presented To	Hypothesised clinical Subscale	Mean scores
1	Have you had frequent bowel movements?	ALL patients	Gut	Relevancy 2.9 Experience 2.3
5	Have you had loose and floating (fatty) stools?	ALL patients	Gut	Relevancy 2.7 Experience 1.1
8	Have you had problems with gas (flatulence)?	ALL patients	Gut	Relevancy 2.9 Experience 2.2
9	Have you had a bloated feeling in your abdomen?	ALL patients	Gut	Relevancy 2.9 Experience 2.1
43	Have you had muscle weakness?	ALL patients	Muscle/energy	Relevancy 2.8 Experience 1.8
44	Have you lacked the energy to do things?	ALL patients	Muscle/energy	Relevancy 3.0 Experience 2.2
12	Have you worried about losing weight?	ALL patients	Weight/food restrictions	Relevancy 2.7 Experience 1.7
19	Have you been restricted in the type of food you can eat due to your bowel movements?	ALL patients	Weight/food restrictions	Relevancy 3.0 Experience 2.1
30	Have you sweated excessively?	ALL patients	Single item	Relevancy 2.9 Experience 1.7
39	Have you felt frustrated?	ALL patients	Single item	Relevancy 2.8 Experience 1.8
6	Have you had dark red or black (tarry) stools?	Gastrinoma/nonfunctioning	Dyspepsia/ulceration	Relevancy 2.6 Experience 1.4
15	Have you had pain in your upper abdomen?	Gastrinoma/nonfunctioning	Dyspepsia/ulceration	Relevancy 2.8 Experience 1.8
13	Have you had acid indigestion or heartburn?	Gastrinoma/nonfunctioning	Dyspepsia/ulceration	Relevancy 2.6 Experience 1.6
47	Have you had itching of the skin?	Gastrinoma/nonfunctioning	Single item	Relevancy 2.6 Experience 1.6
53	Have you had to urinate frequently at night?	Gastrinoma/nonfunctioning	Single item	Relevancy 2.7 Experience 1.8
28	Have you been dizzy?	Insulinoma	Low blood glucose – neurologicalsymptoms	Relevancy 2.9 Experience 1.8
32	Have you had episodes of loss of consciousness?	Insulinoma	Low blood glucose – neurological symptoms	Relevancy 2.9 Experience 1.3
29	Have you felt faint?	Insulinoma	Low blood glucose – neurologicalsymptoms	Relevancy 2.9 Experience 1.7
31	Have you had tremors?	Insulinoma	Low blood glucose – neurological symptoms	Relevancy 2.7 Experience 1.6
37	Have you had problems with your vision?	Insulinoma	Low blood glucose – neurological symptoms	Relevancy 2.9 Experience 1.7
27	Have you experienced hypoglycaemia (low blood glucose)?	Insulinoma	Low blood glucose – other symptoms	Relevancy 3.0 Experience 1.8
18	Have you increased your food intake as a result of your disease or treatment	Insulinoma	Low blood glucose – other symptoms	Relevancy 2.6 Experience 1.5
42	Have you had episodes of confusion?	Insulinoma	Low blood glucose – neurological symptoms	Relevancy 2.6 Experience 1.3
35	Have you felt drowsy?	Insulinoma	Low blood glucose –neurological symptoms	Relevancy 2.8 Experience 1.9

### 3.4 Reliability analyses

Overall results showed for all 24 retained questions together showed α = 0.97 for relevance and α = 0.88 for experience. The results indicate good reliability for the list of retained items and the removal of none of the items result in a substantial change in reliability. Separate internal reliability analyses for the 14 questions for the nonfunctioning and gastrinoma subgroup of patients were also calculated (Cronbach's α relevancy = 0.94; experience = 0.83), and for the 18 questions for the insulinoma subgroup (Cronbach's α relevancy = 0.96; experience = 0.87) again, indicating good internal consistency.

### 3.5 Subgroup analysis of VIPoma and glucagonoma patients

There were very few specific issues related to these tumour subtypes in the literature, and so this data is not reported here. During consultation, interviews with patients with VIPoma (Appendix D) highlighted several overlapping issues that relevant to their quality of life. The most prevalent issue mentioned by patients was chronic diarrhoea, with accompanying lack of energy and fatigue and a related change in appetite (loss) and a feeling of weakness. Patients reported that these symptoms had the most impact on their daily activities, leading to a profound reduction in both physical and social activities. For many patients, it was these changes to their daily activities that were the most difficult to contend with, leading to feelings of anxiety, worry and depression (4 of 8 patients).

The patients with glucagonoma reported feelings of worry, anxiety and/or depression about their condition and/or their symptoms (Appendix D). As with the patients with VIPoma, these feelings were predominantly related to the rarity of their condition, the time to diagnosis, and a more general feeling that there was not as much information available to them as there would be for other conditions. These patients reported alterations in bowel habit, including irregular bowel movements and/or diarrhoea, fatty loose stools, and flatulence, which could change from day to day. A significant number also reported itching and skin rashes as a relevant symptom. Many reported that their most relevant symptom was difficulty in eating, because of issues relating to swallowing, dyspepsia, vomiting and/or reflux. For many this was related to significant weight loss (or difficulties in gaining weight) and constant feelings of hunger, light‐headedness, tiredness, and weakness.

## 4 DISCUSSION

This is the first time that QoL development has been attempted specifically in patients with panNET of any kind. The main themes are not the same as the GINET21, and the questionnaire developed from the data collected in this study is more specific for panNET. This is particularly so for the insulinoma subtype, but data on the gastrinoma/nonfunctioning panNET subgroups are different from those in the GINET21. The findings on reliability for the proposed questionnaires using Cronbach's alpha were good at >0.80, but no further psychometrics were possible on this small group. The results of interviews with patients (glucagonoma/VIPoma) with very rare tumours were important to document since to the best of our knowledge this has never been done before. It was, however, unrealistic to proceed to phase 3 since not enough eligible patients were available for interview with these specific syndromes.

Genetic alterations in panNET^12^ are mainly not present in small bowel NET, showing biological separation of these tumour groups and indicating that new therapies for each may diverge in the future. It has been possible to run international trials on panNET but no specific QoL module has been used.^13^, ^14^ In one of these trials, no QoL measure was used and in the other, the C30 was used and no changes were seen. It is therefore important for specific QoL measurements for this specific, but relatively rare group of tumours.

The limitations of this study are mainly concerning the small cohort studied and the variability of patients being recruited at different stages of treatment, having had different therapies and with different comorbidities. This tumour is rare and in some cases it is challenging to identify patients at the stage where they have significant symptoms but are well enough to undergo interviews, or answer large numbers of questions. It would not be possible to recruit enough patients to stratify for the confounding factors above. The issue of developing modules for patients with rare tumours needs care and possibly slightly different guidelines to common tumours.

For the exceptionally rare syndromes of VIPoma/glucagonoma, some data was gathered and if trials of therapies are planned for these tumours, a symptoms list could be developed using the phase 1 interviews of these patients to assess symptom response.

The new module will be named the PANNET module and will be used alongside the C30 questionnaire in panNET trials. The structure of the module seems best represented by using the C30, followed by the nine generic panNET questions, followed by either five (NF/gastrinoma) or nine (insulinoma) questions. At present, in discussion with the EORTC QoL group, it is suggested the questions are used now as a simple list according to each type of tumour. The subscales are purely clinical and should not be used to aggregate data within them.

Ideally the structure of this module needs to be tested in a phase 4 study. In view of the rarity of the patients, it may be unrealistic to complete a phase 4 study as per the existing guidelines. A validation (phase 4 trial) would normally be done to gain psychometrics to ascertain the final validation of the questionnaire. There will be some issues in identifying an adequate number of patients with each diagnosis, and discussions are ongoing about the feasibility of such a trial. It is likely that some online testing using recruitment of patients via patient support groups may be necessary.

In summary, this study presents the first known data on development of a questionnaire for QoL in panNET. Currently, the list of symptoms can be used for the relevant panNET with the C30 questionnaire to assess response to therapies in this fascinating and diverse group of tumours.

## CONFLICT OF INTEREST

Since the study only involved gathering of data from patients, no conflict of interest is relevant.

## AUTHOR CONTRIBUTIONS

**John Ramage:** Conceptualization, data curation, formal analysis, funding acquisition, investigation, methodology, project administration, validation, writing – original draft, writing – review and editing. **Liz Friend:** Conceptualization, data curation. **Jordan Randell:** Methodology, validation, visualization, writing – review and editing. **Barbara King:** Project administration, supervision, writing – original draft, writing – review and editing. **Paz Fernandez Ortega:** Data curation, investigation, writing – review and editing. **Mairéad Mcnamara:** Investigation, methodology, writing – review and editing. **Gregory Kaltsas:** Investigation; methodology, writing – review and editing. **Massimo Falconi:** Investigation, methodology, writing – review and editing. **Jaroslaw Cwikla:** Investigation, methodology, writing – review and editing. **Jaume Capdevilla:** Investigation, methodology, writing – review and editing. **Simona Grozinsky‐Glasberg:** Investigation, methodology, writing – review and editing. **Dalvinder Mandair:** Investigation, methodology, writing – review and editing. **Eva Gamper:** Formal analysis, investigation, methodology, writing – review and editing. **Rajaventhan Srirajaskanthan:** Data curation, investigation, methodology, writing – review and editing. **Martin O. Weickert:** Investigation, methodology, writing – review and editing. **Debra Gray:** Conceptualization, investigation, methodology, project administration, validation, writing – original draft, writing – review and editing.

## ETHICS STATEMENT

Ethics approval was obtained from UK ethics via Integrated Research Application System and Hampshire Hospitals Foundation Trust was Sponsor of the study (REC16/EM/0245).

### PEER REVIEW

The peer review history for this article is available at https://publons.com/publon/10.1111/jne.13097.

### DATA AVAILABILITY STATEMENT

Source data is available on request from the corresponding author.

## APPENDIX A

### Patient inclusion/exclusion criteria for phases 1‐3.

Inclusion Criteria:

- Grade1‐3 PanNETprimary or metastatic disease and not surgically cured
- Symptoms of Pancreatic Neuroendocrine Tumour
- Able to understand the language of the questionnaires
- Aged 18 years or above
- Absence of any psychological or physical condition potentially hampering compliance with the study protocol
- Capable of understanding the study patient information sheet and providing written informed consent in accordance with ICH GCP and national/local regulations and procedures

Exclusion Criteria:

- Prior curative surgery for pNET with no current disease
- Assisted in the Phase 1 or Phase 3 interviews for the development of the original QLQ‐GINET21 module
- Under 18 years of age
- Unable to understand the language of the questionnaires
- Patients who are unable to self‐complete QoL questionnaires or participate in a structured oral interview where the QoL questionnaires are administered by a researcher or clinician
- Presence of a psychological or physical condition which would make it difficult for them to comply with the protocol or provide written informed consent
- Participating in other QoL investigations that might interfere with this study
- Have another concurrent cancer, other than localised skin cancer.

## APPENDIX B

### Phase 3 Demographic Data, Karnofsky status and grade of tumour.

**Table:**

	Gastrinoma	Insulinoma	NF PanNET
Age Mean (range)	61 years (45‐70)	54 years (24‐83)	67 years (44‐85)
Gender	Male=5 Female=12	Male=9 Female=10	Male=16 Female=7
Karnofsky Status Score mean (10‐100%)	92%	84%	85%
ENETS Grade. Numbers in each grade (1‐3) N/A not available	G1=7 G2=5 G3=1 N/A=4	G1=11 G2=4 G3=1 N/A=3	G1=9 G2=9 G3=1 N/A=4

## APPENDIX C

### List of excluded items with reasons for exclusion.

**Table:**

Item	Item	Reason for Exclusion	Mean scores
2	Have you had to move your bowels at night?	Low average relevancy scores. Less than 50% of sample scored 3 or 4 for this item.	Relevancy: 2.2 Experience: 1.3
3	Have you felt the urge to move your bowels quickly?	Low average relevancy scores. Less than 50% of sample scored 3 or 4 for this item.	Relevancy: 2.5 Experience: 1.9
4	Have you had blood in stools?	Low average relevancy scores.	Relevancy: 2.6 Experience: 1.1
7	Have you worried that you might be incontinent (stool)?	Low average relevancy scores. Less than 50% of sample scored 3 or 4 for this item.	Relevancy: 2.5 Experience: 1.6
10	Have your daily activities been limited by your bowel problems?	Low average relevancy scores.	Relevancy: 2.6 Experience: 1.6
11	Has weight gain been a problem for you?	Was considered to be non‐specific and could be measured objectively.	Relevancy: 2.7 Experience: 1.8
14	Have you had acid reflux?	Low average relevancy scores. Less than 50% of sample scored 3 or 4 for this item.	Relevancy: 2.5 Experience: 1.4
16	Have you had problems swallowing?	Low average relevancy scores. Less than 50% of sample scored 3 or 4 for this item.	Relevancy: 2.3 Experience: 1.2
17	Have you had a problem with regurgitated food coming into your mouth?	Low average relevancy scores. Less than 50% of sample scored 3 or 4 for this item.	Relevancy: 2.4 Experience: 1.2
20	Have you changed your diet as a result of your disease or treatment?	Was seen to be relevant, but it was not clear if it should be positively or negatively scored.	Relevancy: 3 Experience: 2.3
21	Have you felt hungry?	Low average relevancy scores	Relevancy: 2.6 Experience: 1.7
22	Has your appetite increased?	Low average relevancy scores	Relevancy: 2.5 Experience: 1.7
23	Have you needed to get up at night to eat?	Low average relevancy scores. Less than 50% of sample scored 3 or 4 for this item.	Relevancy: 2.3 Experience: 1.2
24	Have you had problems with your sense of taste?	Low average relevancy scores. Less than 50% of sample scored 3 or 4 for this item.	Relevancy: 2.4 Experience: 1.6
25	Have you had soreness in your mouth?	Low average relevancy scores. Less than 50% of sample scored 3 or 4 for this item.	Relevancy: 2.3 Experience: 1.3
26	Have you had pain in your mouth?	Low average relevancy scores. Less than 50% of sample scored 3 or 4 for this item.	Relevancy: 2.3 Experience: 1.2
33	Have you had palpitations?	High relevance but low experience scores. Not clinically relevant.	Relevancy: 2.9 Experience: 1.6
34	Have you had confusion or unusual behaviour at night?	High relevance but low experience scores. Not clinically relevant.	Relevancy: 2.7 Experience: 1.2
36	Have you had problems with your coordination?	High relevance but low experience scores. Found to be non‐specific	Relevancy: 2.7 Experience: 1.4
38	Have you had problems speaking clearly?	High relevance but low experience scores. Found to be non‐specific	Relevancy: 2.7 Experience: 1.3
40	Have you felt anxious?	Item already covered in C30 questionnaire	Relevancy: 2.8 Experience: 1.9
41	Have you or others noticed a change in your behaviour?	High relevance but low experience scores. Found to be non‐specific	Relevancy: 2.8 Experience: 1.7
45	Have you felt lethargic?	Similar to item 44, removed for duplication.	Relevancy: 2.9 Experience: 2
46	Have you had a skin rash?	Low average relevancy scores	Relevancy: 2.4
48	Have you worried that your skin or eyes are yellow?	Low average relevancy scores	Relevancy: 2.4 Experience: 1.2
49	Have you had swollen legs or ankles?	Low average relevancy scores	Relevancy: 2.6 Experience: 1.5
50	Have you had pain in your back?	Low average relevancy scores	Relevancy: 2.7 Experience: 1.9
51	Have you had pain in the area of surgery?	Low average relevancy scores	Relevancy: 2.4 Experience: 1.4
52	Have you had headaches?	Low average relevancy scores	Relevancy: 2.8 Experience: 1.6
54	Have you had hot flushes?	Low average relevancy scores	Relevancy: 2.6 Experience: 1.5
55	Have you been satisfied with the information you have received (e.g. about	Narrow range of scores. Ceiling effects.	Relevancy: 3.7 Experience: 3.5
56	Have you felt you have adequate support (e.g. from family, friends, healthcare professionals)?	Narrow range of scores. Ceiling effects.	Relevancy: 3.8 Experience: 3.7
57	Have you felt less interest in sex?	Patients found this item intrusive and not relevant in debriefing interviews.	Relevancy 2.7 Experience: 1

## APPENDIX D

### Phase 1 Issues for Glucagonoma/VIPoma Patients.

**Table:**

VIPOMA symptoms	Prevalence= (n of patients reporting issue, n=8)
Diarrhoea (chronic)	8
Lack of Energy, tiredness and/or Fatigue	5
Appetite change (loss)	5
Weakness	4
Anxiety/Worry/Depression	4
Abdominal pain	3
Nausea and/or Vomiting	3
Back Pain	2
Difficulty Sleeping	2
Weight Loss	2
Double vision	2
Shortness of breath	2

**Table:**

Glucagonoma Symptoms	Prevalence (number of patients reporting the issue n=10)
Anxiety/Worry/Depression	7
Bowel Alterations	6
Lack of Energy, tiredness and/or Fatigue	6
Weight Loss	6
Difficulty Eating	5
Dyspepsia (indigestion)	5
Skin Rash/Itching	5
Light‐headedness	4
Vomiting	4
Abdominal Pain	3
Feeling weak	2
Difficulty Sleeping	2
Nausea	2
Change in Taste	2
